# MA-RTI: Design and Evaluation of a Real-World Multipath-Assisted Device-Free Localization System

**DOI:** 10.3390/s23042199

**Published:** 2023-02-15

**Authors:** Marco Cimdins, Sven Ole Schmidt, Fabian John, Manfred Constapel, Horst Hellbrück

**Affiliations:** 1 Department of Electrical Engineering and Computer Science, Technische Hochschule Lübeck, Mönkhofer Weg 239, 23562 Lübeck, Germany; 2 Fraunhofer Center for Maritime Logistics and Services, Blohmstraße 32, 21079 Hamburg, Germany

**Keywords:** device-free localization, multipath-assisted, radio tomographic imaging, ultra-wideband, channel impulse response, system architecture, raytracing, multipath components, MQTT

## Abstract

Device-free localization (DFL) systems exploit changes in the radio frequency channel by measuring, for example, the channel impulse response (CIR), to detect and localize obstacles within a target area. However, due to a lack of well-defined interfaces, missing modularization, as well as complex system configuration, it is difficult to deploy DFL systems outside of laboratory setups. This paper focused on the system view and the challenges that come with setting up a DFL system in an indoor environment. We propose MA-RTI, a modular DFL system that is easy to set up, and which utilizes a multipath-assisted (MA) radio-tomographic imaging (RTI) algorithm. To achieve a modular DFL system, we proposed and implemented an architectural model for DFL systems. For minimizing the configuration overhead, we applied a 3D spatial model, that helps in placing the sensors and calculating the required calibration parameters. Therefore, we configured the system solely with idle measurements and a 3D spatial model. We deployed such a DFL system and evaluated it in a real-world office environment with four sensor nodes. The radio technology was ultra-wideband (UWB) and the corresponding signal measurements were CIRs. The DFL system operated with CIRs that provided a sub-nanosecond time-domain resolution. After pre-processing, the update rate was approximately 46 Hz and it provided a localization accuracy of 1.0 m in 50% of all cases and 1.8 m in 80% of all cases. MA fingerprinting approaches lead to higher localization accuracy, but require a labor-intensive training phase.

## 1. Introduction

A DFL system detects, tracks, and identifies objects, such as persons, within the target area. In comparison to device-based systems, DFL systems do not require an electronic device in order for a person to be detected, localized, or identified. We envision DFL systems for deployment in real-world environments to support applications, such as elderly care, ambient assisted living, intrusion detection, and smart-home application where one or multiple persons in buildings and rooms need to be detected and tracked with a localization accuracy of about 1–2 m to determine the room and the rough position of the person in the room.

The term device-free passive localization was introduced, in 2007, by Youssef et al. [1]. However, we use the shorter term device-free localization (DFL) for the rest of this article, as device-free and passive is considered redundant.

Literature, like that in [2], distinguishes different kinds of DFL systems, such as camera-based or acoustic- and ultrasonic-based systems. Camera-based localization relies on the acquisition of high-resolution images, which imposes completely different challenges, such as computationally intensive signal processing, high data rate demands, and privacy concerns. Acoustic and ultrasonic signals have different propagation characteristics and frequencies compared to RF signals.

In our work, we focused on DFL systems in the radio frequency (RF) domain by means of IEEE 802.15.4a UWB networks. RF-based DFL systems offer the advantage of combining both device-free localization and regular data transmission (the necessary signal measurements are performed during message reception). IEEE 802.15.4a, in particular, is already being used for indoor positioning systems. For a comprehensive overview and evaluation of the strengths and weaknesses of different technologies for DFL, refer to [2]. In addition, Alam et al. provide an excellent overview of non-RF-based DFL systems in [3]. In this paper, we developed a modular online DFL system with low configuration and installation effort. For communication between the required function blocks, we proposed a distributed and scalable architecture, based on the message queuing telemetry transport (MQTT) protocol for RF-based DFL systems, that can also be implemented within infrastructure networks, i.e., based on IEEE 802.11 or IEEE 802.15.4(a).

RF-based DFL systems measure and process the change within the RF signal propagation to detect, track, and identify one or multiple persons within a target area. Detection is the identification of changes within the target area, according to [1]. Tracking is the continuous determination of the position of a person within a target area. Identification is a task to determine the type (human, non-human), size, mass, and shape of a person [1].

To implement the functionality for detection, tracking, and identification, a signal variation is required, based on propagation effects, such as reflection, shadowing, scattering, and diffraction. The signal strength is measured e.g., with RSS. Therefore, DFL systems transmit messages or use existing ones, such as beacons, and record the RSS on receipt. The measurements are processed in order to detect and track persons within the target area. Besides RSS, radio technologies, such as IEEE 802.11, offer the complex-valued CSI of orthogonal frequency division multiplex (OFDM) radio communication. The CSI represents the complex values for each amplitude of the sub-carrier of the radio transmission, and, thus, provides the DFL parameter over various frequencies.

UWB communication systems, such as IEEE 802.15.4a, designed for indoor localization systems, offer a complex-valued CIR in time domain. The fine-grained radio measurements improve the localization accuracy and reliability of DFL systems.

Another challenge for DFL systems in indoor environments is multipath propagation leading to constructive and destructive interference of the received signals. Ignoring multipath propagation effects reduces localization accuracy. To cope with multipath propagation, measurements over multiple frequencies, e.g., multiple channels, the CSI, or deploying of UWB systems, are proposed [4,5,6,7].

Another recent approach is to exploit multipath propagation. Considering multipath propagation increases localization accuracy or reduces the number of physical sensors, as shown in [7,8,9].

**Figure 1:**
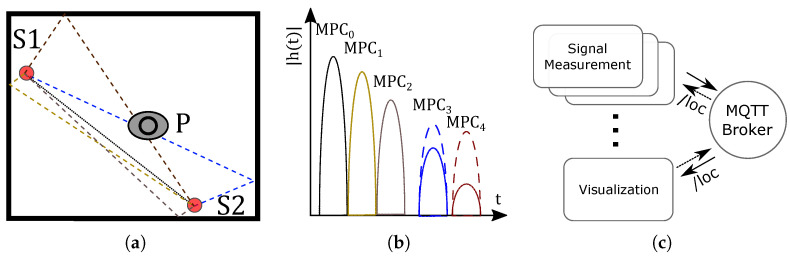
Principle of the proposed MA DFL System and an excerpt from the system view. (**a**) Map information with MPCs. (**b**) Resulting UWB CIRs. (**c**) Modular system.

Our main motivation in writing this paper was that we are not aware of online DFL systems deployed in productive real-world environments, as only a few works have considered a system view of DFL. Figure 1 depicts the principle of our proposed multipath-assisted DFL system and provides an excerpt of the system view, ultimately providing an overview of the contributions of this paper. Assume two wireless sensor nodes, S1 and S2, which are deployed in a room (see Figure 1a). The transmitted signal arrives at the receiver on the direct path (${\mathrm{MPC}}_{0}$) and via echo paths that are reflections on the walls (${\mathrm{MPC}}_{1},\ldots ,{\mathrm{MPC}}_{4}$). Figure 1b depicts the resulting UWB CIR, which is composed of the different MPCs. A person standing at position *P* affects ${\mathrm{MPC}}_{3}$ and ${\mathrm{MPC}}_{4}$ and, therefore, alters the magnitude of the MPCs in comparison to the idle case, where no person is present. The dashed lines indicate the magnitude of the altered MPCs. A novelty of multipath-assisted (MA) DFL systems is that they can break down alterations from different MPCs. We used a simple 3D spatial model that extracted the map information to map the altered MPCs to the target area.

In this paper, we propose a modular system that is composed of function blocks and the communication between each function block takes place via the MQTT protocol. Figure 1c shows the processing chain, composed of signal measurement, signal processing, and localization algorithm. The localization result is published to the topic /loc, and subscribed by the visualization. In this paper, we propose and implement the system configuration, data acquisition, signal processing, localization algorithm, and visualization. Our contributions are as follows:

- We present a novel architectural model for building a distributed, multipath-assisted (MA) DFL system. The main focus is on distributed and lightweight communication between functional blocks and the use of map information for system construction.
- We describe and implement the architectural model using a multipath-assisted DFL system. The UWB CIR measurements are extracted from an IEEE 802.15.4a UWB network.
- MA radio tomographic imaging (RTI) has shown promising results in the past. However, the measurements were presented in an outdoor scenario. We present a means by which to transfer MA-RTI to indoor environments, with a simple 3D spatial model of the room, and we evaluate the overall system. In addition, we compare the training-free MA-RTI with a MA fingerprinting approach, MAMPI, that incorporates feature vectors based on magnitude and phase differences for localization.

The rest of the paper is structured as follows: Section 2 presents the principle of DFL and provides an overview of multipath-assisted DFL systems, together with real-world implementations. In Section 3, we propose a simple architectural model for DFL systems. We describe the implementation of an MA DFL system that is based on UWB CIRs in Section 4. The proposed system is evaluated in Section 5. Finally, in Section 6, we conclude this paper and give an outlook for future work.

## 2. Principles of Device-Free Localization and Related Work

DFL systems consist of electronic devices that emit signals that are measured in return. Typically, the electronic devices are wireless transceivers within a network, named sensor nodes in this paper. Note: As stated beforehand, persons that are to be detected and tracked do not wear any device that participates in the process of localization. The emitted signals are often regular packets sent within the network e.g., beacons of wireless APs. The information about the presence of a person is contained in the electromagnetic field strength, measured by a channel measurement. Typically, a channel measurement is an RSS measurement, e.g., the RSSI value, the energy detection value, or the fine-grained CSI, or CIR. We refer to a periodic measurement of the channel measurement stream (sometimes called link in literature). As stated in the introduction, we focused on channel measurements that are measured during wireless network communication.

The working principles, a generic and layered three-tier system architecture, and the most relevant channel measurements are presented in [10]. However, following the classification of DFL systems from [11], DFL systems can be fingerprint-based or stream-based.

### 2.1. Fingerprint- and Stream-Based DFL Systems

A fingerprint-based DFL system requires a training phase in order to create a passive radio map. In contrast with radio maps that are recorded with active (e.g., smartphone-based) systems, we use the term passive radio map from [1]. The passive radio map is created by placing a person in pre-defined reference positions and measuring the channel measurement for the positions. Throughout this paper, we use the term reference position. Other authors use cells, zones, and locations in this context. During an online phase, the passive radio map is used to compare the current measurements with the trained ones in order to determine the position of the person. Examples of fingerprint-based systems are [1,6,12,13,14]. A person stands at a reference position, while the sensor nodes perform the channel measurements and save them as a fingerprint. The fingerprint-based DFL system does not require the positions of the sensor nodes in order to locate the person. To overcome the tedious training phase, Zhou et al. proposed a ray-aided generative adversarial model for the construction of the passive radio map [15].

In contrast to fingerprint-based systems, stream-based DFL systems evaluate changes in channel measurements in any stream. A person moving within the target area affects the channel measurement of the streams. These changes are detected and processed to localize persons. Typically, stream-based algorithms employ a calibration phase to estimate channel measurements of each stream, while the target area is vacant. Examples of stream-based systems are [16,17]. Characteristics such as the mean and the variance for each stream describe the state of each stream.

Extensive training phases increase the installation and maintenance effort of DFL systems. Therefore, our system was calibrated solely with idle measurements, i.e., measurements done while the target area was vacant. We performed such calibration phases after placing the sensor nodes and could repeat them when no person was present within the target area e.g., at night.

### 2.2. Multipath-Assisted DFL Systems

As stated before, DFL systems must cope with multipath propagation, otherwise the multipath propagation decreases the localization accuracy. Some authors avoid multipath by measuring the channel measurement over various channels in order to combine the information, or pick the channel that is least affected by multipath [4,5,18].

In [19], Schmidhammer et al. showed that multipath propagation could be exploited actively for DFL. In [20], the authors demonstrated, by calculating theoretical performance bounds, how the coverage of the target area increased by including MPCs for DFL. While in [19] the authors deployed a channel sounder. They deployed low-cost commercially off-the-shelf (COTS) UWB radio chips in [21]. COTS-available UWB radio chips enable access to UWB CIR measurements, where multiple complex-valued MPCs can be extracted from one measurement [7,9,22]. In addition to the magnitude of the MPCs, the phase of the complex-valued MPC can be modeled and exploited in MA-DFL systems [8,23]. Furthermore, UWB CIRs enable bi- and multi-static radar approaches that are able to localize a person by extraction of additional signal paths [24,25,26]. However, these approaches are again susceptible to multipath propagation [9]. In this work, we proposed applying an MA-RTI approach to our real-world indoor localization system. MA-RTI is solely calibrated with idle measurements and a 3D spatial model. In contrast, we compared the localization accuracy of MA-RTI with an MA fingerprint approach, MAMPI, that required an extensive training phase [7,22].

### 2.3. Exploitation and Transport of Channel Measurements

In [27], the problem of human motion detection in IEEE 802.11 (WiFi) networks was targeted by measuring body-induced alterations of RF signals. The data collection and signal processing for extraction of the Channel State Information (CSI) was done on a dedicated device. Subsequently, the CSI was serialized using JavaScript Object Notation (JSON) and transmitted to any client application with the message queuing telemetry transport (MQTT) application protocol utilizing a publish/subscribe pattern. In our work, we focused on sensing with IEEE 802.15.4(a), while deploying a more distributed approach.

The proposed platform for real-time processing and analytics in [28] used different types of channel quality information (CQI) to support passive detection and tracking of objects with radio sensing and vision technologies. The architecture consisted of multiple nodes. Any node collected CQI values and pushed them to a gateway. The gateway performed low-level feature extraction and forwarded the features to the cloud for real-time detection, classification, and tracking. All data processed in the cloud was made accessible via representational state transfer (REST) in JSON format for client applications, such as localization and behavior recognition. Our approach did not rely on cloud services, so it was computationally less expensive.

An architecture with ensemble models trained with machine learning for counting people with CSI in dense IEEE 802.11 infrastructure networks was proposed and evaluated in [29]. However, the proposed system design depended on WiFi devices with multiple antennas, and MIMO techniques. In our work, we did not require MIMO features, since we did not count, but rather tracked, objects.

In [30], a DFL system with real-time capability for occupancy sensing with commercial off-the-shelf (COTS) WiFi devices was presented. First, CSI was collected on an IoT platform. Subsequently, the transformation of the CSI values to human presence and CSI time series data to human activity information was done on a cloud server, aided with a MQTT broker. Clients (e.g., smartphones) subscribed topics on the MQTT broker to collect data for application purposes. Similar to our approach, the data transport was implemented via MQTT. However, we used MQTT for modular, bi-directional communication via several topics, as described in the following section.

## 3. System Design

In this section, we present the main contribution of our paper, namely, a scalable and modern system design for DFL systems with a focus on ease of integration. After a brief description of the conceptual shape of any DFL system, with the aid of function blocks, we take an established DFL system architecture, partitioned in responsibility planes, and link them together to yield our proposed system design.

### 3.1. DFL Function Blocks

**Figure 2:**
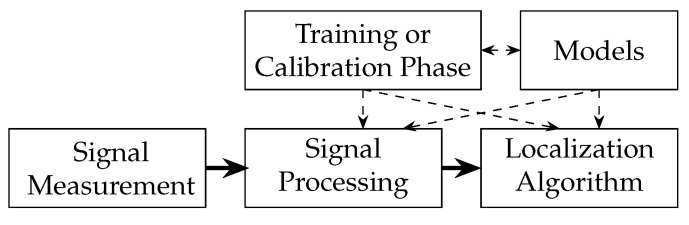
General function blocks of a DFL system.

In general, a DFL system consists of common parts that we name function blocks [12,14]. These function blocks operate in a processing chain. They comprise signal measurement, signal processing, localization algorithm, training or calibration phase and models. A DFL system requires a function block that performs the signal measurements as shown in Figure 2. The first block measures one or several channel measurements as raw data that is processed further in the rest of the system. Due to noisy measurements, and to extract desired features from the raw data, signal processing is performed in the next step. The localization algorithm extracts information about the presence and position of one or several persons. A training or calibration phase is needed to create reference data to better distinguish between multipath effects and the influence of the person on the channel measurement. The results of this training or calibration are used in the online phase by the localization algorithm. Models are the bases to interpret the measurements, detect multiple persons, predict movement, and simulate the RF propagation. In Figure 2 thick lines indicate data that is processed fast during runtime, while dashed lines indicate configuration parameters that are required to control the processing blocks.

Signal Measurement: The signal measurement function block is the source of the channel measurements. It creates and formats the measurements e.g., RSS, CSI, or CIR measurements. This measurement block is dependent on wireless networking technology. Examples for IEEE 802.11-based DFL systems are [1,12,31]. Abdel-Nasser et al. used the CSI of the IEEE 802.11n in [6]. IEEE 802.15.4 was the technology used in [32,33,34]. Proprietary sub-GHz DFL systems were proposed in [11,35]. UWB CIR were proposed in [7,24,36,37,38].

Signal Processing: The next block is the signal processing function block that processes the raw data and prepares the data for localization algorithms. Examples are filters or the calculation of the mean or the variances of each stream e.g., in [12,16,31]. Other systems use the α-trimmed mean filter e.g., in [14] or detection of stream outliers e.g., in [14,33]. Anomaly-based DFL systems normalize the result in order to detect whether there is a person near the affected stream (see [16,31,34]). UWB CIR measurements are often time-aligned [39] and sinc-interpolated [7]. From the CIR, the complex-valued MPCs can be extracted [23], or the CIR can be processed as a whole [8,24].

Localization Algorithm: Finally, the localization algorithm function block estimates the hidden state, such as the presence or position of the persons. The state information changes slowly compared to the measurement signal, so fast processing beyond this block is not required anymore. However, the demand for flexibility and extensibility increases, so that state fusion with other systems could be integrated.

Training or Calibration Phase: All DFL systems have either a training phase to record the fingerprints and to map the position of a person to a certain reference position, or a calibration phase to adjust parameters, such as the mean or variance of RSS values, while the target area is vacant. This block mainly instructs the measurement block of the data plane.

Models: For the processing and algorithms, the modeling block provides propagation models and other models, e.g., mobility models, that control the underlying processing or localization blocks. Previously proposed are, e.g., person models, such as conditional random field (CRF) [11,14], hidden Markov model (HMM) [14] or person motion models [16]. Thereby, the block also validates and corrects the processing of the measurements and position estimations, e.g., by adding spatial and temporal constraints.

### 3.2. Architectural Model

**Figure 3:**
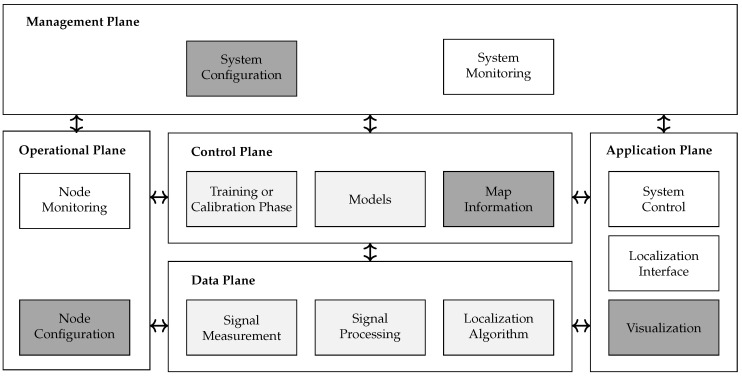
Proposed architectural model. The light gray-filled blocks within the control and data plane are DFL function blocks, covered in research within the last decade. The dark gray-filled function blocks were proposed and implemented in this work. The non-filled function blocks are the subject of current research.

As a base for our system design, we incorporated the architecture that was proposed in the context of software-defined networking (SDN) consisting of five planes [40], as shown in Figure 3: data, control, management, operational and application.

To enhance existing wireless networks for DFL, the DFL system requires an extension of firmware that is able to supervise the state of the sensors and either able to perform the signal measurements itself or extract the data from received network packets. Further, in this paper, we demonstrate how this is achieved with a UWB network. The data has to be forwarded to a server that processes and runs the algorithms. Additionally, a block for operation and maintenance needs to be added. Consequently, the proposed architectural model included the following well-known DFL function blocks: signal measurement, signal processing, localization algorithm, training or calibration phase, and models, as well as additional blocks for managing the network.

In the following, we develop and explain the tasks and functions of the proposed architectural model, plane by plane, starting with the data plane.

Data Plane: The data plane is also called the forwarding plane in SDN. An important aspect of the data plane is the efficient and fast processing of data. Consequently, channel measurements, such as RSS, CSI, and CIR, that require fast processing are handled in this plane. In general, all DFL system function blocks that process the data in such a manner are placed here. These are signal measurement, signal processing, and localization algorithms.

Control Plane: The control plane instructs or configures the data plane on how to process the data. In the networking domain, firewall and routing functionality are placed in the control plane to generate rules for forwarding, filtering, and intercepting packets in the data plane. In DFL, the data plane behavior is controlled by configuration parameters, which are created from a training or calibration phase, models, and the map information.

Map Information: Maps are an additional valuable source of information for controlling the algorithms of the data plane. They provide entry and exit points for persons to the target area and check whether the calculated path of algorithms is reasonable (e.g., persons cannot walk through walls). The blocks in the control plane should continuously adapt to changes in the environment. Research in the last decade has focused on these two planes and the light gray highlighted blocks mainly. However, real-world systems need additional planes for operations, which are operational, management, and application planes.

Operational Plane: The operational plane contains functionality that operators of the network devices require (e.g., for restarting or shutting down a device). In DFL systems, we place functions to switch measurement on and off, for processing, and for localization or load, and replace configuration parameters or models in the control plane. We differentiate here between configuration and monitoring functionality, e.g., node monitoring as operators need to monitor as well as configure the network device. For instance, battery-powered sensors have to go into a sleep state in order to save energy according to their energy level in the battery.

Management Plane: The task of the management plane is to monitor, configure and maintain one or more network devices or parts of network devices. It may also configure the forwarding plane, but it does so infrequently. For DFL systems, we introduce the function blocks system configuration and system monitoring. System configuration can, for example, identify the network nodes that are suited for DFL. Furthermore, the blocks in the plane are responsible for organizing the structure of the system and assigning network nodes to cells. The idea of the management plane is to create an interface for the system operator to access, for example, a group of devices, such as WiFi APs or other sensors, on a system level. In real-world applications, it is necessary to control sensors or control and coordinate a training phase. For these tasks, a dedicated management application, or an interface, is required.

Application Plane: Applications and services that use services from the control and/or management plane form the application plane. The task of the application plane is to provide a localization interface and visualization of the localization, and to create an interface for system control. The localization interface enables the localization results to be accessed by external applications connected to the DFL system. External applications include enterprise resource planning (ERP)-systems or safety applications. The visualization shows, for example, the number and location of persons and highlights their paths. Finally, the system control provides the system operator with an API to access all settings of the system.

### 3.3. Our DFL System Design

Our system design took the five architectural planes and included the function blocks. The model included all function blocks of DFL systems, as well as blocks and functionality that we proposed for integration of DFL systems in existing networks, which are needed for real-world deployments. Some blocks in this proposed architecture, as well as the communication and interfaces between the different planes, remain an open issue for future work.

#### 3.3.1. MQTT-Based Transport Network

The message queuing telemetry transport (MQTT) protocol was designed to be open and easy to implement [41]. It utilizes a scalable and event-driven publish/subscribe architecture that supports thousands of clients from a single server, called the MQTT broker. The MQTT broker is responsible for creating and maintaining the message link between publishers (data producers) and subscribers (data consumers). This architecture enables applications with weak dependencies between publishers and subscribers. In general, MQTT is ideal for usage in constrained environments with high latency or low bandwidth, respectively, as well as on embedded systems with limited memory and processing capabilities, which are critical aspects of Internet of Things (IoT) applications [42,43]. Furthermore, in contrast to HTTP with its document-centric request/response paradigm, MQTT permits lower power consumption and less protocol overhead [44]. We decided in favor of MQTT, instead of REST-based protocols, such as HTTP or Constrained Application Protocol (CoAP), because of the publish/subscribe pattern. The main advantage of the publish/subscribe pattern is that each function block only needs to know the broker address. In addition, once data is published (e.g., the UWB CIRs with its metadata), multiple subscribers can subscribe to this topic and receive the data. There is no need to poll the data by multiple sources and cache it.

**Figure 4:**
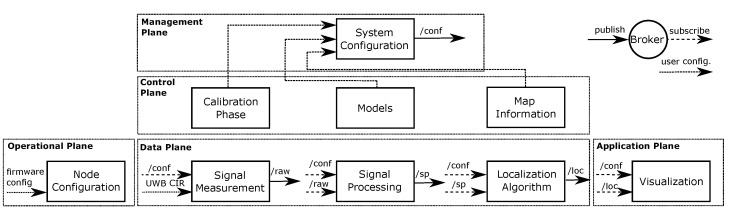
The DFL system design implements a publish/subscribe pattern for message exchange and inter-process communication. The function blocks, except the node configuration, are MQTT clients, that publish and subscribe data from the MQTT broker. The control plane provides information for the system configuration.

Figure 4 presents the proposed system. The central data distribution unit of our system was the broker that connected all the function blocks. The arrows between the planes indicate the flow of data or control messages. Dashed lines indicate that data was subscribed from the broker, and solid lines indicate that data was published to the broker. The narrow dashed lines indicate user configurations that were required for the system configuration.

The UWB CIR measurements are published as raw data via the MQTT topic /raw. The signal processing function block subscribes to the topic /raw, performs calculations on the data (refer Section 4.3.1), and publishes its results in /sp. Further, the localization algorithm uses the processed data and publishes the calculated result to the topic /loc. With these topics, we create the processing chain in the data plane that processes the channel measurement toward the localization algorithm. Although the function blocks are logically connected, MQTT enables a distributed execution of the function blocks over various machines. Furthermore, the system can run in parallel with multiple instances that are configured, e.g., with different parameters.

#### 3.3.2. Scheme of Operation

The system is configured and calibrated in the initialization and calibration phases. Models provide parameters for the signal processing and map information provides the boundaries of the room. The system configuration block gathers the information and creates the system configuration object, which is published to the topic /conf. All function blocks subscribe to the /conf topic and extract their relevant configuration parameters.

Finally, the systems’ data are visualized in the visualization block. This block subscribes the configuration to display, e.g., the map information, and shows the localization results.

As the sensor nodes are not connected to the MQTT broker (they solely communicate via the UWB radio), the node configuration is done within the firmware of the sensor nodes. The broker is installed locally on a laptop. The function blocks of the data plane are processes that subscribe data from the broker, perform the calculations and publish their results back to the broker. Therefore, each block of the data plane processes the signal measurement, signal processing, and localization algorithm. The results are visualized in another process. The calibration phase subscribes to the pre-processed UWB CIRs and determines the relevant parameters. The 3D spatial model is a separate program. The system configuration collects all the required information and calculations and publishes the configuration in the /conf topic. As the algorithms are lightweight, the broker and the processes run locally on a laptop. In the calibration phase, when the system is offline, we compute the required parameters described in (14). As long as the network configuration does not change [4], the system just multiplies the new measurement vector z with the matrix result from (14). For details, refer to Section 4.3.3. If required, due to, for instance, higher processing demands, we could use an external MQTT broker and run the data plane within the cloud.

## 4. Implementation

In this section, we provide the implementation details for our real-world MA DFL system. We describe each required function block in their corresponding planes and highlight the design decisions.

### 4.1. Map Information

In this paper, we designed and evaluated a MA DFL system. MA DFL systems exploit both the direct path between each sensor, and echo paths coming from reflections, such as walls. Exploiting multipaths helps in reducing the number of required sensors or increases the accuracy of the DFL system. Therefore, we describe the map information block of our proposed system. In order to reduce the training effort, we deployed a simple 3D spatial model that helped in finding good placements for the sensor nodes and for calculation of reflection paths and their respective time delays $\tau_{i}$. Specifically, we placed the sensor nodes, performed raytracing, and extracted the required information for calibration of the system.

#### 4.1.1. Including Multipath Handling in the System

To set up an MA DFL system, we had to exploit multipath propagation. Therefore, we briefly describe the handling of multipath propagation and channel impulse responses in this section, as UWB CIRs enable the extraction of multiple MPCs from one CIR.

The transmitter Tx sends a UWB pulse x(t) that is received by the receiver Rx. The pulse arrives at the receiver in a direct path (Tx,Rx) and, via additional echo paths, due to reflections on walls and other obstacles. Echos of x(t) on the *i*-th path are received with a time delay $\tau_{i}$ and an altered amplitude $a_{i}$. The CIR h(t) represents the multipath propagation of all *I* signal echos between Tx and Rx:

(1) $$\begin{array}{c} h\left(t\right)=\sum_{i=0}^{I-1}a_{i}\delta_{0}\left(t-\tau_{i}\right), \end{array}$$

where $\delta_{0}\left(t\right)$ is the Dirac function.

The received signal y(t) at Rx is a superposition of all received signal echoes:

(2) y(t)=x(t)∗h(t)+n(t),

where n(t) is additive white Gaussian noise [45].

**Figure 5:**
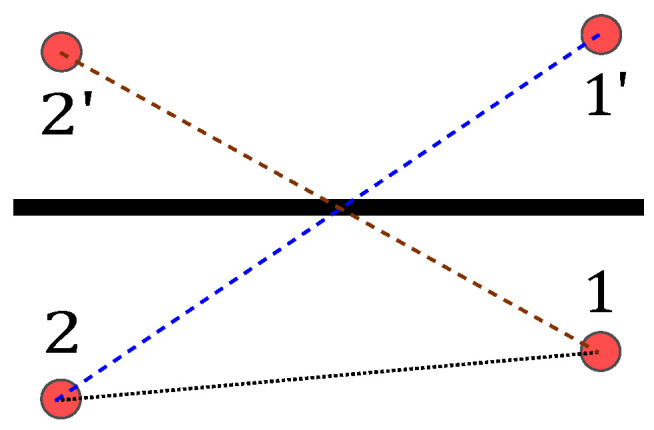
Direct and echo path of UWB transmission.

Figure 5 shows a multipath scenario with one reflective surface, e.g., a wall (bold black line).

To enrich RTI with multipath, every sensor position is mirrored on each wall. This results in virtual sensor positions of $1^{\prime }$ and $2^{\prime }$ for each wall. As an input vector for RTI, we used the permutations of the physical and virtual sensor pairs $\left(1,2^{\prime }\right)$, $\left(1^{\prime },2\right)$, and the direct path (1,2) $\left(\mathrm{here}:\;{\mathrm{MPC}}_{0}\right)$. For each reflection on a wall (incident and reflected ray), we gained two MPCs that would be assigned the same MPC value. The difference to conventional RTI systems is that the former only exploit the direct path. The MA approach adds two additional paths for each wall that creates a reflection in the system.

The power of the incoming UWB signal is divided at each reflection [46]. Depending on the signal’s angle of arrival, and the material of the reflector, parts of the UWB signal are transmitted through the reflector, and the remaining part is reflected. This transmitted portion is also referred to as reflection loss. The measurement environment of this work consisted mainly of walls, which provided a good reflection behavior for UWB signals, due to low reflection losses for all angles of incidence. Since, in the presented method, MA-RTI only required the difference between two received signals, the exact amount of the reflection loss was negligible.

For the *i*-th MPC, the measurement vector for the *i*-th MPC $z_{i}=z\left[i\right]$ is defined as:

(3) $$z_{i}=\left|P_{\mathrm{MPC},i,\mathrm{obs}}-P_{\mathrm{MPC},i,\mathrm{idle}}\right|,$$

where $P_{\mathrm{MPC},i,\cdot }$ is the signal power of the observed and idle CIR at $\tau_{i}$, respectively [9].

When a person affects an MPC, either on the direct or an echo path, in comparison to an idle target area, MA-RTI assigns pixels in the proximity of the signal paths a high attenuation value. As in previous work, we used the absolute difference for each MPC as input for our system (see (3) [9]).

#### 4.1.2. Three-Dimensional Spatial Model

To include the influence of multipath propagation for MA-RTI, we created a simple 3D spatial model of a room and performed raytracing. As shown in [47], a suitable raytracing model was able to estimate the CIR well enough for our purpose. The developed software was structured in multiple steps.

1. Construction of the 3D spatial model: To construct a 3D spatial model, we analyzed the general structure of the room. Based on a self-selected origin, we provided distinctive points, such as corners of the room or larger pieces of furniture, with their respective coordinates in *x*-, *y*- and *z*-dimensions. These points served as vertices for the spatial model. The corresponding walls of the structure (including horizontal walls e.g., ground or ceiling) were defined as surfaces, delimited by a subset of the set of defined vertices. At least three vertices are required to delimit a surface, whereas real walls (as in our given office environment) generally have at least four vertices. By definition of the surfaces, the 3D spatial model was described fully.

To keep the model applicable, we required centimeter–decimeter accuracy of the determined coordinates. We welcomed a higher accuracy, but omitted this for practical reasons. Typically not all walls have a perfectly smooth surface, whiteboards attached to the wall and other structures, such as door frames, stick out 1–2 cm. The effort required to model all objects was too high. In addition, the UWB CIR in this work resolved with a time-domain resolution of approximately of 1 ns, and, therefore, it could only resolve MPCs that were 30 cm apart from each other. After defining the room’s geometry, we placed the sensor nodes on their respective coordinates.

2. Calculation of the Echo Paths: To map MPCs to the target area, we determined the paths of all valid important signal echo paths of the setup. Note: In the following, each node can represent both the Tx and Rx with respect to whether it is the sensor’s turn to transmit or to receive.

**Figure 6:**
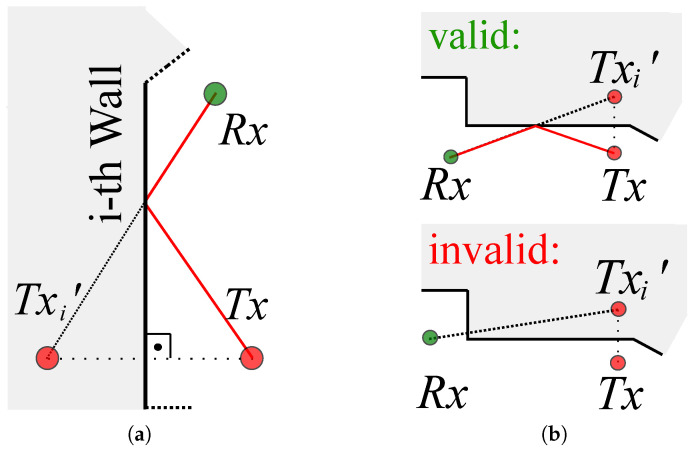
Modeling of raytracing. (**a**) Determination of the *i*-th virtual node $Tx_{i}^{\prime }$. (**b**) Valid and invalid echo paths.

This general procedure is shown in Figure 6. First, the node that represents Tx was mirrored three-dimensionally on each wall of the room (see Figure 6a). For this purpose, the vertical line on the respective wall that ran through the coordinates of Tx was determined. The resulting route between the wall and Tx was continued in the same direction and length behind the wall. The end of this route then corresponded to the virtual node $Tx^{\prime }$. For (I−1) walls, we calculated (I−1) virtual nodes $\left\{Tx_{i}^{\prime }\right\}$.

Not all virtual nodes $Tx^{\prime }$ lead to reflection of the signal and, thus, to a valid echo path. Figure 6b shows an example of a valid path and an invalid path. For each virtual node $Tx_{i}^{\prime }$, we defined a flag $f_{val,i}$. Then, the virtual node $Tx_{i}^{\prime }$ was linked to Rx. The echo path was valid when the intersection of this signal path was inside the delimited surface of the corresponding 3D spatial model’s wall. In this case, the corresponding flag was set to $f_{val,i}=1$. When the intersection did not fit this condition, the flag was set to $f_{val,i}=0$. Finally, all signal paths with set flags $f_{val,i}=1$ were valid, and the remaining ($f_{val,i}=0$) were neglected for further analysis. The intersection with the wall was geometrically identical to the reflection point of the signal echo path. So, each path was describable unambiguously by the start position Tx the reflection point, and the end position Rx.

**Figure 7:**
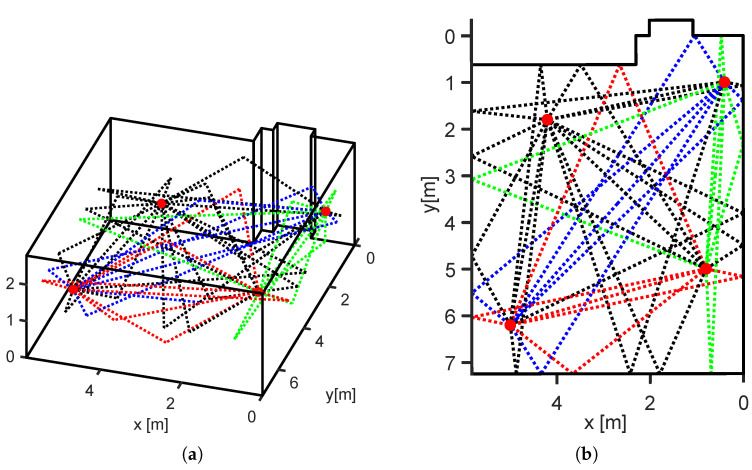
Raytracing for evaluation setup. (**a**) Three-dimensional room geometry of the evaluation setup with MPCs. (**b**) Top view of the 3D room geometry with MPCs.

Figure 7 depicts all valid 1st-order echo paths for the room geometry of our test environment, including four sensor nodes. Note: multiple walls on the northern side of the room structure did not result in valid signal echo paths.

3. Determination of the Transmission Delays: To extract the MPCs of the CIR, we needed to determine the transmission delays $\tau_{i}$ of the *i*-th MPC, i=1,…,I−1. Due to geometry, the length of the *i*-th echo path was the distance between the virtual node $TX_{i}^{\prime }$ and Rx. Since the coordinates of both positions were known, the distance was calculated using the Euclidean distance. With the speed of light $c_{0}$, the transmission delay $\tau_{i}$ follows:

(4) $$\begin{array}{c} \tau_{i}=\frac{d_{i}}{c_{0}}=\frac{||Rx-Tx_{i}^{\prime }\left|\right|}{c_{0}}, \end{array}$$

where ||·|| is the Euclidean distance.

Note: As described, a 3D spatial model is required to determine the valid MPCs and the corresponding $\tau_{i}$ for the permutation of the sensor pairs. Options for calculating the MPCs were hand-written programs, commercial RF propagation tools, or open-source programs, such as proposed in [48].

### 4.2. Signal Measurement

The task of the signal measurement function block is to extract and aggregate the channel measurement and forward it logically to the signal processing block.

Note: all function blocks are connected to the MQTT broker, where the extracted and aggregated channel measurements are published into the topic. This enables, on the one hand, subscribing by the signal processing function block and, on the other hand, other functions, such as logging by subscribing to the same topic.

There are two approaches for the extraction of the channel measurement: In infrastructure networks, sensor nodes may be connected directly to the MQTT broker e.g., via WiFi. In this case, we require an API that enables publishing the channel measurement directly to the broker. Furthermore, not all sensor nodes that measure a channel measurement have an additional WiFi radio, due to energy or cost constraints.

#### 4.2.1. Node Configuration

In this work, we set up the system with a different approach. In order to utilize multipath propagation for DFL, we placed sensor nodes equipped with an UWB radio chip in the target area. In the firmware of the sensor nodes, we read out the UWB CIR and the required metadata. On receipt of a UWB message, the CIR, which was the channel measurement, was measured and saved. To propagate the measured UWB CIRs, the data was contained inside the exchanged messages. One sensor node acted as a listener, and listened to the UWB radio communication, extracted the UWB CIR measurements, and forwarded the data to the broker.

#### 4.2.2. Signal Measurement with DW1000 Firmware for UWB CIR Measurements and MQTT Adapter

Table 1 provided the payload of the exchanged UWB messages. The payload contained metadata (first 26 Byte), followed by the complex CIR values.

The binary serialized metadata and CIR data were sent within a CIR frame via one listener node in the DFL system, which was connected to a computer via a serial communication interface. On the computer, the received CIR frame was forwarded as a MQTT message to the topic /raw. The payload for messages published to the topic /raw was still binary, which increased the performance of further processing blocks. Binary serialization and deserialization avoided computationally expensive parsing and rounding errors. Note: serialization and deserialization on different hardware architectures require handling of the correct byte order.

To interpret the received serial buffer, the binary serialized structure had to be known. Therefore, necessary information, such as the length of the metadata and the cir, were stored as parameters in the global /conf topic.

### 4.3. Signal Processing

The signal processing block processed the raw data from the signal measurement and prepared the data for the localization algorithm.

#### 4.3.1. Pre-Preprocessing of UWB CIR

For our proposed MA-RTI system, we had to process the raw UWB CIR measurements and extract the magnitude of each MPC. Therefore, we increased the resolution of the CIR by a suitable sinc-interpolation. Furthermore, we aligned the CIRs in time. After subscribing to the raw UWB measurements, we received the payload described by Table 1 in Section 4.2.2. After deserialization, the data would be processed further.

In the following, we briefly summarize the pre-processing we proposed in [7]:

The raw CIR $h_{\mathrm{raw}}\left(kT_{s_{1}}\right)$ was a series of *K* I/Q values with k=0,1,…,K−1. The bandwidth of the IEEE 802.15.4.a channel was B=499.2 MHz. This resulted in a CIR that was sampled with $T_{s_{1}}\approx 1$ ns. Within the firmware, we aimed to measure a CIR with a maximum length of approximately 50 ns, and, therefore, we set K=50 samples.

We resampled the raw CIR with $T_{s_{2}}$ and applied the sinc-interpolation. This resulted in a fine-grained CIR $h_{\mathrm{IP}}\left(nT_{s_{2}}\right)$ (n=0,1,2,…).

To align the CIRs in time, we used the timestamp from the leading edge detection of the Decawave DW1000. The timestamp was provided on an integer value with an accuracy of approximately 1 ns and a fine-grained fractional part that narrowed the timestamp down in steps of 1/64 ns. Our firmware saved and transmitted the CIRs’ five samples before the reported integer part. By the combination of the time alignment and the protocol, which captured the UWB CIRs, we reduced possible synchronization errors.

After sinc-interpolation and time alignment, we scaled the CIR with its reported $P_{\mathrm{Rxlvl}}$, which was given by (6). Then, we cropped each CIR to exactly *N* samples, resulting in $h(kT_{s_{2}})$, with n=0,1,…,N−1. For detailed information and example source codes for the pre-processing, refer to [7].

#### 4.3.2. Extraction of MPCs

In the following, we describe the extraction of MPCs from the pre-processed CIR $h(kT_{s_{2}})$:

Equation (5) provided the magnitude of the MPC in dBm [49]:

(5) $$P_{\mathrm{MPC}}=10\;\log_{10}\left(\frac{F_{-1}^{2}+F_{0}^{2}+F_{1}^{2}}{N_{\mathrm{cnt}}^{2}}\right)-A_{\mathrm{PRF}},$$

where $F_{-1}$, $F_{0}$, and $F_{1}$ are the magnitude values of the CIR at time $\left\{t_{\mathrm{MPC}}-1\;\mathrm{ns},t_{\mathrm{MPC}},t_{\mathrm{MPC}}+1\;\mathrm{ns}\right\}$. $N_{\mathrm{cnt}}$ was the preamble accumulation count, and the constant $A_{\mathrm{PRF}}=121.74$ dB was valid for the pulse repetition rate of 64 MHz [49].

$P_{\mathrm{Rxlvl}}$ in dBm was calculated as [49]

(6) $$P_{\mathrm{Rxlvl}}=10\;\log_{10}\left(\frac{C\cdot 2^{17}}{N_{\mathrm{cnt}}^{2}}\right)-A_{\mathrm{PRF}}.$$

To process the extracted MPCs for MA-RTI, we determined the input vector z as follows:

(7) $$z[i]=z_{i}$$

(8) $$=|P_{\mathrm{MPC},i,\mathrm{obs}}-P_{\mathrm{MPC},i,\mathrm{idle}}|$$

For this, we extracted for the idle reference CIR and the observation CIR the power of each MPC $P_{\mathrm{MPC}}$ with (5).

Note: the complex-valued CIR enabled the extraction of the phase for each MPC with the following equation [7]

(9) $$\phi =\arctan\left(\frac{I\{h\left(kT_{s_{2}}\right)\}}{R\{h\left(kT_{s_{2}}\right)\}}\right)\;\left[\mathrm{rad}\right].$$

After extraction, we unwrapped ϕ and determined the phases at the corresponding MPCs. Then we unwrapped ϕ and read the phases at the corresponding MPC positions. ${\mathrm{MPC}}_{0}$ typically carried the information of the direct link between Tx and Rx. To compensate for unknown phase offset, we calculated the relative phase $\Delta \phi_{i}$ of the *i*-th path using [7]:

(10) $$\Delta \phi_{i}=\phi_{i}-\phi_{0}$$

#### 4.3.3. Localization Algorithm: MA-RTI

The localization algorithm function block subscribed the result from the signal processing and estimated the position of the person.

In the following, we briefly introduce the principle of RTI. For detailed information that includes, for example, figures for illustration of the weight function (12), refer to [9]. The target area *A* was divided into *J* equally sized pixels. RTI determined the attenuation value for each pixel, based on the measurement. The vector v represented the heat map. The matrix W of size I×J assigned weights $w_{ij}$ to the heat map. z was the measurement vector of length *I*, with *I* being the number of the direct and echo paths (in our MA case, all the valid 1st-order MPCs of all sensor pairs (see Figure 7a,b)).

RTI systems are based on a simple linear model [17]:

(11) z=Wv+n,

where n is *I*-dimensional normally distributed noise.

We determined the weight $w_{ij}$ for each path *i* and each pixel *j* with the following equation [9,17]:

(12) $$w_{ij}=\left\{\begin{array}{cc} 1/\sqrt{d_{i}} & \mathrm{if}\;d_{ij}\left(1\right)+d_{ij}\left(2\right)<d_{i}+\lambda \\ 0 & \mathrm{otherwise} \end{array}\right.,\;$$

where $d_{ij}\left(1\right)+d_{ij}\left(2\right)$ is the distance from Sensor 1 to Sensor 2 on the *i*-th path over the center of pixel *j*. $d_{i}$ is the distance of the *i*-th path, and λ is a tuning parameter in $R^{+}$ [9,17].

Solving (11) with L2-minimization was an ill-posed inverse problem, which required regularization of the pseudo-inverse with a covariance matrix of *v*$C_{v}$ weighted by $\sigma_{J}^{-2}$. The elements of the covariance matrix were defined as in [9,17]:

(13) $$C_{v}\left[k,l\right]=\sigma_{v}^{2}e^{-d_{kl}/\delta_{c}},$$

where $d_{kl}$ is the distance from pixel *k* to *l*, $\delta_{c}$ a space constant, and $\sigma_{v}^{2}$ is the pixel variance of the estimation error.

Including the covariance matrix in the calculation resulted in:

(14) $$\hat{v}={\left(W^{T}W+C_{v}^{-1}\sigma_{J}^{2}\right)}^{-1}W^{T}z$$

We determined the estimated position of the person ${\hat{r}}_{P}$ by searching the maximum value in $\hat{v}$ [9]:

(15) $${\hat{r}}_{P}=\mathrm{Pos}\left(\underset{j\in \{0\ldots J-1\}}{arg max}{\hat{v}}_{j}\right),$$

where operator Pos(j) returned the position vector of the *j*-th pixel.

**Figure 8:**
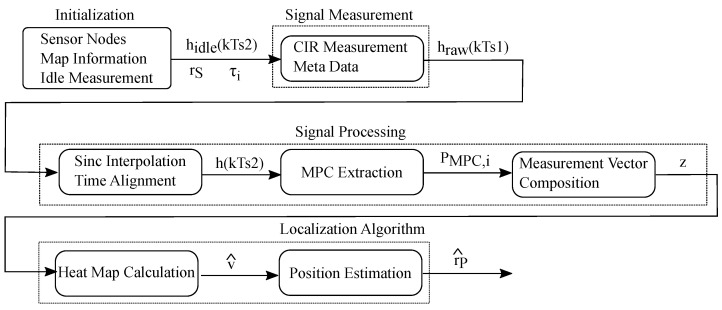
Block diagram of the localization algorithm.

Figure 8 shows the block diagram for the localization algorithm MA-RTI. After initialization, we recorded the raw UWB CIRs $h_{\mathrm{raw}}\left(kT_{s_{1}}\right)$, together with the metadata. The raw CIRs would be sinc interpolated and aligned in time $h_{\left(kT_{s_{2}}\right)}$, then the corresponding signal values $P_{\mathrm{MPC},i}$ at the respective MPCs $\tau_{i}$ would be extracted. All signal values from valid MPCs formed the measurement vector z which was required for heat map calculation $\hat{v}$ and position estimation ${\hat{r}}_{P}$, by finding the position of the pixel that had the maximum value.

Next to the estimated position ${\hat{r}}_{P}$, we published the heat map $\hat{v}$ to the MQTT broker. Then, the visualization function block visualized the estimated position and the heat map.

#### 4.3.4. Localization Algorithm: MAMPI

In the following, we briefly provide the details for a fingerprinting approach MAMPI that we used for comparison. For detailed information, refer to [7].

Assume the target area *A* contained *P* positions. We referenced each position with the position indicator $r_{p}$. For each of the person’s positions $r_{p}$ with p=1,⋯,P, we determined a reference feature vector $s_{p}$. $s_{p}$ served as a fingerprint and contained overall $L_{s}$ values to characterize $r_{p}$. In addition, we recorded an observation feature vector $s_{o}$ that contained a person at an unknown position.

MAMPI exploits the magnitudes and phase differences that are extracted from the UWB CIR to form the feature vector [7]:

(16) $$s_{p}=\left(\begin{array}{c} P_{\mathrm{MPC}0,p}\\ \cdots \\ P_{\mathrm{MPC}I-1,p}\\ \Delta \phi_{1,p}\\ \cdots \\ \Delta \phi_{I-1,p} \end{array}\right).$$

For localization, we determined the similarity of $s_{o}$ to the reference feature vectors $s_{p}$ of all positions *p*. To do so, we calculated the $\ell_{1}$-norm $d_{\ell_{1}}$ [7]:

(17) $$d_{\ell_{1}}\left(s_{o},s_{p}\right)=\sum_{l=1}^{L_{s}}\left|s_{o}\left(l\right)-s_{p}\left(l\right)\right|.$$

To determine the likeliest position ${\hat{r}}_{p}$ as position estimation, we followed the nearest neighbour approach. The best fitting estimate minimized the calculated $\ell_{1}$-distance $d_{\ell_{1}}\left(s_{o},s_{p}\right)$ of all positions *p* [7]:

(18) $${\hat{r}}_{p}=\arg\;\min_{p}\left(d_{\ell_{1}}\left(s_{o},s_{p}\right)\right).$$

### 4.4. Test Setup

In the following, we describe the test setup for the evaluation of our proposed system.

#### 4.4.1. MQTT Broker

We selected Mosquitto v.2.0.15. as an MQTT broker. Although other MQTT brokers were available, the performance of local MQTT brokers was comparable to each other [50]. The broker ran locally on a laptop with Windows 10, i7-6600U, 16 GByte RAM.

In the future, we will deploy an MQTT broker connected to the Internet and connect the sensor node that acts as the listener on a small computer to forward the raw values to this publicly available broker. Then, we will run the signal processing and localization on a server and log the data for long-time evaluation.

#### 4.4.2. Scenario

**Figure 9:**
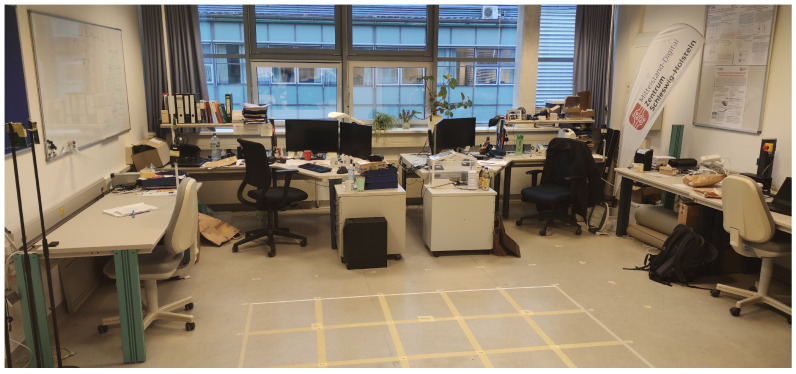
Photograph of the room.

We set up our proposed MA-RTI system in a typical office building room that provided a multipath-rich environment. Figure 9 provides a photograph of the room.

The size of the room was approximately 6 × 7 m. One side contained a window front, the other walls were various tables and superstructures. The center of the room was kept free for the measurement. We attached the sensors to the ceiling with a holder.

**Figure 10:**
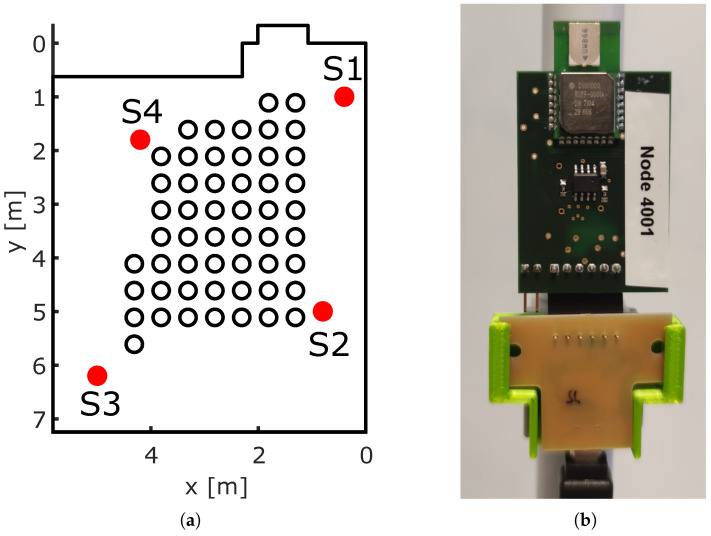
Theoretical MPCs calculated by raytracing. (**a**) Room with reference positions. (**b**) Sensor node plattform uLoc.

Figure 10a provides the top view of the room, together with the sensor and reference positions. We chose the reference positions to cover the majority of the room, while keeping approximately 0.5 m distance from obstacles, such as tables. The reference positions were 0.5 m apart from each other.

We placed four sensor nodes at a height of 1.418 m, which was half the height of the room. The sensor nodes were mounted magnetically from the ceiling with PVC tubes. The magnetic holder allowed the sensors to be moved easily. The (x,y)-coordinates of the sensors were as follows: Sensor 1 was placed at (0.4,1) m, Sensor 2 at (0.8,5) m, Sensor 3 (5,6.2) m, and Sensor 4 at (4.2,1.8) m.

#### 4.4.3. Sensor Nodes and Radio Settings

Figure 10b shows a photograph of our sensor nodes uLoc. The uLoc utilized a Decawave DWM1000 UWB radio module that operated the Decawave DW1000 UWB radio chip. The sensor node was controlled by an ATXMega128A1.

We adapted the firmware in [9] to utilize four sensor nodes. Additionally, another sensor node acted as the listener. The listener listened to the UWB radio communication and forwarded the transported CIR measurements via a serial interface to a computer that forwarded the data toward the MQTT broker. The radio settings of the Decawave DW1000 were as follows: We utilized IEEE 802.15.4.a channel 3 with a center frequency of 4.4928 GHz. The bandwidth *B* for the chosen channel was 499.2 MHz. The pulse repetition frequency was set to 64, the preamble length to 128, and the preamble acquisition chunk size to 8. The Tx and Rx preamble code was 9, and the nodes transmitted with a data rate of 6.8 MBit/s. As the frames that transported the CIR, together with the metadata, were 242 Byte, we set the PHY header mode to extended-length data frames.

In the following, we provide more information about the Decawave DW1000 UWB radio chip. The DW1000 supported 6 RF bands from 3.5–6.5 GHz, with channel bandwidths of 500–900 MHz. The typical output power spectral density was programmable and its typical value was −39 dBm/MHz [51]. The cost of the used DWM1000 module was about 10–20 $. Due to the bandwidth of approximately 500 MHz, the time-domain resolution of the CIR was approximately 1 ns. With proper time alignment, using the fractional part of the leading edge detection, the time-domain resolution was improved to sub-nanosecond accuracy (see Section 4.3.1).

### 4.5. Calibration Phase

During the calibration phase, we recorded 5000 CIR frames, while the target area was idle. Based on the idle measurements, we determined the idle magnitudes $P_{\mathrm{MPC},i}$ for each MPC. To enable fast processing for the localization algorithm, we calculated the terms in front of the measurement vector z from (14), namely the covariance matrix $C_{v}$ and the weight matrix W. The calibration results were sent to the system configuration functional block that published the results on the /conf topic.

## 5. Evaluation

In this section, we evaluate our proposed DFL system.

### 5.1. Measurements of CIR Frame and Localization Rate

To evaluate the throughput of our proposed live system, we measured the raw CIR frame rate and the localization rate during the operation of the system. For this, we subscribed to the respective MQTT topics and logged the timestamps during transmission. Namely, for the CIR frame rate, we subscribed on the topic /raw, and for the localization rate, we subscribed on the topic /loc. For evaluation of the localization algorithm, refer to Section 5.3.

The CIR frame rate was mainly dependent on the firmware of our nodes. In 3623 s we measured 167,965 CIR frames, which resulted in a CIR frame rate of 45.99 Hz.

The localization rate depended on the CIR frame rate and the number of frames that were required for localization. In 3652 s our system calculated 7880 localization estimations, resulting in a localization rate of 2.16 Hz.

In previous work, we evaluated the performance of a local MQTT broker for devices connected via WiFi or Ethernet. Depending on the provided bandwidth of the network connection, we achieved a message throughput of more than 5000 msg/s for payloads between 100–1000 Bytes [52]. Therefore, we assumed that the message throughput would be sufficient for further setups.

In the following, we discuss possible improvements for future work: Currently, we transmit the raw CIR measurements together with the metadata. One CIR frame has 242 Bytes of data, which could be reduced significantly if the signal processing took place directly on the sensor nodes. Parameters required for extraction of the MPCs, especially the expected $\tau_{i}$, could be published in a separate MQTT topic and sent to the sensor nodes via UWB from the listener.

The following approaches would improve the localization rate: Our firmware measures and transmits the CIR from Tx to Rx and vice-versa. For localization, we currently only utilize the CIR measurements from Tx to Rx. Using both values significantly reduces the number of required CIR frames required for localization. Furthermore, our localization algorithm waits for a batch of CIRs measurements and then performs the localization. In the future, we will implement and evaluate either a sequential or sliding window-based processing, and, therefore, update the heatmap each time a CIR frame arrives.

### 5.2. Comparison of Simulated with Measured MPCs

We set up and calibrated the system solely with a simple 3D spatial model and idle measurements. Our test setup reassembled a typical office, filled with furniture and other objects that were not modeled in the 3D spatial model (see Figure 7a). Note: modeling furniture and other objects is possible but it increases the installation effort of the system. In the following, we evaluated the system calculated $\tau_{i}$, required for extraction of the MPCs within the CIR measurements, although many obstacles were not modeled.

**Figure 11:**
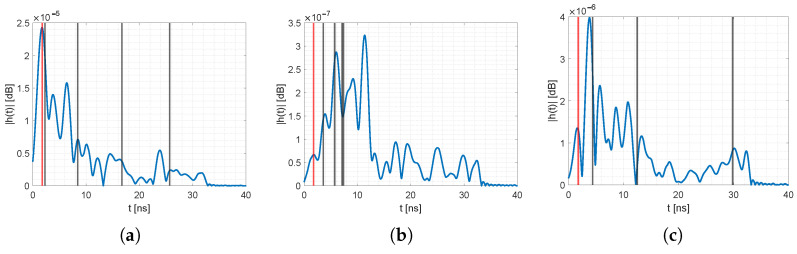
Idle CIRs for different sensor pairs. (**a**) CIR with MPCs from (1,2). (**b**) CIR with MPCs from (1,3). (**c**) CIR with MPCs from (1,4).

**Figure 12:**
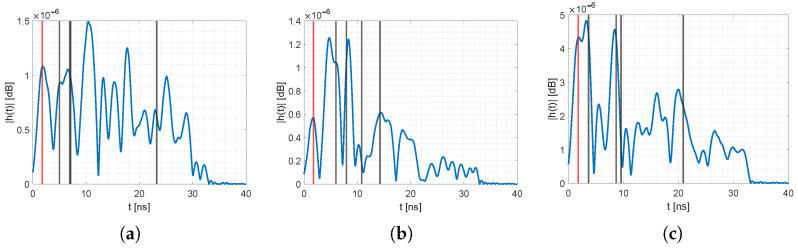
Idle CIRs for different sensor pairs. (**a**) CIR with MPCs from (2,3). (**b**) CIR with MPCs from (2,4). (**c**) CIR with MPCs from (3,4).

Figure 11 and Figure 12 provide the reference CIRs for all unique permutations of the sensor nodes, together with the position of the MPCs $\tau_{i}$ extracted from raytracing of our 3D spatial model. The blue line is the pre-processed idle reference CIR. The vertical lines represent the *i*-th MPC at $\tau_{i}$, determined from the 3D spatial model. The red line is the direct path (${\mathrm{MPC}}_{0}$), and the black line represent MPCs from the other walls. Note: Although the ground and ceiling reflection affected the CIR, we did not extract those values; however, they might interfere with neighboring MPCs [53]. For all the idle CIRs, the MPCs were either at a peak or in close proximity. Deviations might come from inaccuracies of the 3D spatial model, e.g., unmodeled furniture, placement of sensor nodes that were a few cm apart from the ground truth position, or interference from neighboring MPCs. Nevertheless, the resolution of the 3D model was enough to extract the relevant MPCs with sufficient accuracy. We saw similar deviations of the MPCs in an outdoor scenario. For an evaluation of the MA-RTI algorithm in an outside environment, refer to our previous work in [9].

### 5.3. Localization Results

We calculated the Euclidean distance to evaluate the localization accuracy:

(19) $$e=\left|\right|{\hat{r}}_{p}-r_{p}\left|\right|$$

where ||·|| is the Euclidean distance, ${\hat{r}}_{p}$ the estimated position, and $r_{p}$ the ground truth position of the person.

For each sensor pair, we determined a reference idle value $h_{\mathrm{ref}}\left(t\right)$ from the idle measurements that had its $P_{\mathrm{Rxlvl}}$ close to the mean of $P_{\mathrm{Rxlvl}}$ [8].

Differing from previous work [9], where we calculated the mean CIR from 100 measurements, we took single snapshot measurements. In our case, we waited for 20 CIR frames, extracted the MPCs, and calculated $\hat{v}$.

For each of the 53 reference positions shown in Figure 10a, we recorded 2300 CIR frames. To access localization accuracy, we batch-processed 100 position estimates from 20 CIR frames at a time.

For the proposed MA-RTI algorithm, we used the following parameters, which were close to the values we deployed in our previous work [9]: We divided the target area into pixels with a distance of 0.1 m, the width of the weighting ellipse was λ=0.01 m, and we set the pixel variance $\sigma_{v}^{2}$ and the regularization parameter $\sigma_{J}^{2}$ to $0.5\;{\mathrm{dB}}^{2}$. The pixel correlation constant was increased from $\delta_{c}=0.5$ m to $\delta_{c}=0.7$ m.

**Figure 13:**
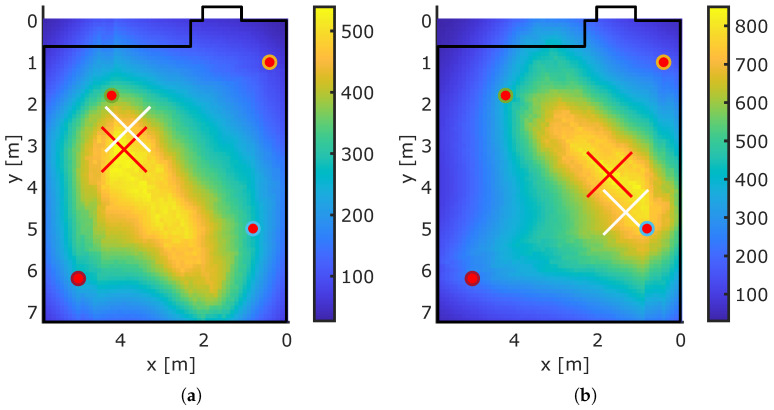
Exemplary MA-RTI results. (**a**) Exemplary Pos. 1. (**b**) Exemplary Pos. 2.

**Figure 14:**
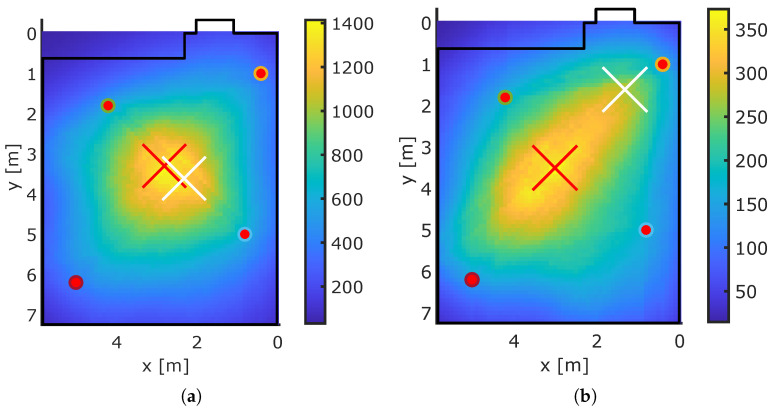
Exemplary MA-RTI results. (**a**) Exemplary Pos. 3. (**b**) Exemplary Pos. 4.

Figure 13 and Figure 14 show exemplary localization results for our MA-RTI system. The red dots are the sensor nodes, the white cross indicates the ground truth position $r_{p}$, and the red cross indicates the estimated position ${\hat{r}}_{p}$. The bold black lines indicate the walls of the room, extracted from the 3D spatial model. The color map depicts the heatmap v of MA-RTI: Positions that are dark blue have a low probability of the person being located at that pixel, and a bright yellow color indicates a high probability.

The ground truth position in Figure 13a was close to the estimated position. The person affected many MPCs in the CIR of (1,4) and (3,4). The ground truth position in Figure 13b was close to Sensor 2. The position estimate tended toward the middle of the room because many MPCs of different sensor pairs were affected that passed through those pixels. When the person was close to the middle of the room (see Figure 14a), the position estimation was close to the ground truth position. Figure 14b the person stood at a position that was mostly covered by MPCs from two sensor nodes (1,3). As the MPCs crossed mostly the middle of the room, the maximum of the heatmap was located there, resulting in large localization errors.

For the localization algorithm MAMPI, we divided the data measured at the 53 reference positions into training and test data seta. Different from machine learning applications, that typically use 80% of the data for training and 20% for evaluation [54], we used 10% of the data for training and the rest for evaluation as in [7]. We used the mean of the training set for each position as reference fingerprints.

**Figure 15:**
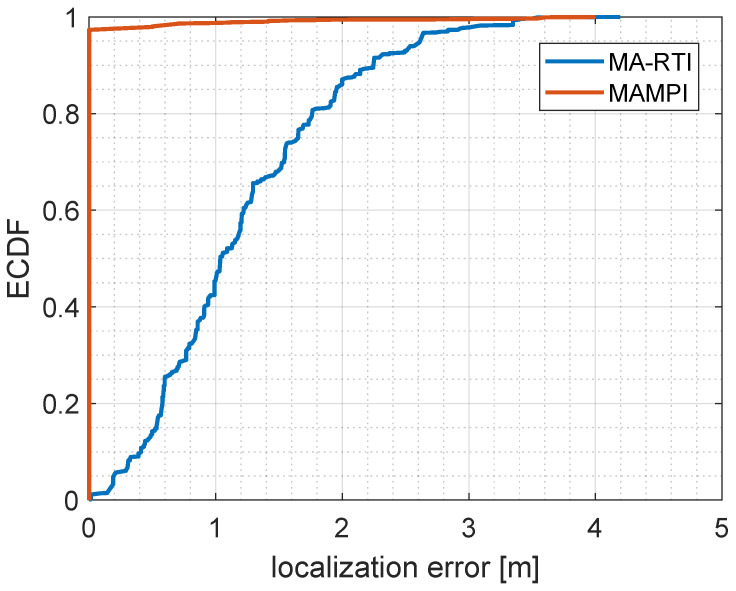
Photograph of the room.

Figure 15 provides the empirical cumulative distribution function (ECDF) for all localization results for MA-RTI (blue line) and MAMPI (red line). From the ECDF we determined the 50% and 80% percentile: For MA-RTI, we achieved a localization error below 1.0 m in 50% of the cases, and a localization error below 1.8 m in 80% of the cases. Positions that were in the vicinity of several MPCs had a localization accuracy of less than 1 m. This was sufficient for our envisioned applications of DFL systems, although still subject to further improvement. In the future, we will improve the weight function and systematically evaluate the information content for each MPC to gain insight on an optimal sensor node placement and improved localization results. Furthermore, we will investigate solutions based on $\ell_{1}$ minimization, as proposed in [55]. In comparison to MA-RTI, the MA fingerprinting approach MAMPI estimated the correct position in 97% of all cases. This was due to the fact that we evaluated with data where a person was standing at the exact positions that we used for training. We expected that the fingerprints would deteriorate over time and that the localization error would increase when the person qas not standing in proximity of a trained reference position [56]. Still, fingerprinting provided high localization accuracy with the main drawback of the tedious training phase that had to be repeated or updated after changes in the environment. Future research includes the automatic creation of the passive radio map, e.g., by ray-tracing [15], or by applying a radio propagation model, as proposed in [13].

### 5.4. Discussion

In this paper, we proposed a DFL system based on an architectural model. To connect the different function blocks and to build a modular distributed system, we proposed the MQTT architecture. MQTT implements a publish and subscribe pattern and enables simple access to required values by publishing configuration parameters and measurement data. MA DFL requires map information to determine the virtual sensor positions and to extract the information for MA-RTI. We showed how to extract this information with a simple 3D spatial model and raytracing. This enables easy calibration of the system, together with idle measurements that are performed when the target area is idle. We achieved a high CIR frame rate of approximately 46 Hz that could be significantly increased in the future, when the signal processing is implemented directly on the sensor nodes. The localization rate was better than 2 Hz, which is subject to improvements. Our goal was to develop a DFL system with a minimum number of sensors. To cover the target area with a sufficient amount of MPCs, we placed four sensor nodes that were equipped with a UWB radio in the target area. With four sensors, we could localize positions that were in the vicinity of several MPCs with an accuracy of less than 1 m. In addition, with a more realistic weight function for MA-RTI (such as proposed in [18]), the localization accuracy would be increased in the future.

Fingerprint approaches, such as MAMPI, result in higher localization accuracy. In our case, where the person stood at the same positions that were trained, we estimated the exact position in 97% of the cases. However, we expect that this accuracy would deteriorate over time and the effort of the training phase prevents large-scale distributions of fingerprint approaches.

The proposed and implemented online and modular system would help to improve the function blocks one by one, as one could immediately see the changes. Furthermore, the publish/subscribe pattern enables subscribing to the topics and implementing/providing results for different algorithms without higher effort. The following steps must be performed to transfer the MA-RTI system to a new environment, e.g., a different room: The 3D spatial model has to be adapted for the new room and idle measurements recorded for calibration. We expect the localization accuracy to remain in the same order of magnitude as long as all objects are in the 3D model significantly responsible for multipath propagation.

## 6. Conclusions and Future Work

In this paper, we proposed an architectural model for real-world DFL systems. Furthermore, we proposed MQTT for communication and configuration of the function blocks. Based on the architectural model, we set up an MA DFL system that localized a person within a typical office room, with UWB CIR measurements. To reduce installation and maintenance costs, we configured and calibrated the MA DFL system with a simple 3D spatial model and idle measurements. The system provided a CIR frame rate of approximately 46 Hz and localization update rate of approximately 2 Hz. We achieved a localization accuracy of 1.0 m in 50% of the cases and of 1.8 m in 80% of the cases with our setup. If higher localization accuracy is required, MA fingerprinting approaches, such as MAMPI, may lead to lower localization errors. However, fingerprinting, in general, requires an extensive training phase that we were aiming to avoid with our training-free approach.

The future work includes evaluations of other radio technologies in different setups, i.e., installing the system in different rooms. Improving the localization accuracy of MA-RTI, by replacing the RTI weight function and accessing the information content for each extracted MPC, is still work in progress. Furthermore, we will implement and evaluate other localization algorithms while having the system in operation. In this regard, our focus lies on algorithms based on $\ell_{1}$ minimization.

## Figures and Tables

**Table 1:** CIR payload adapted from [9].

Variable	Type	Meaning
srcID	uint16_t	ID of the source node
nodeID	uint16_t	ID of the originating CIR measurement
intPart	uint16_t	integer part required for time alignment
fracPart	uint16_t	fractional part required for time alignment
maxNoise	uint16_t	maximum noise level
stdNoise	uint16_t	standard deviation of noise level
maxGrowthCIR	uint16_t	max. value
rxPreamCount	uint16_t	preamble count $N_{\mathrm{cnt}}$
fppl	double	$P_{\mathrm{MPC},0}$
rxlvl	double	$P_{\mathrm{Rxlvl}}$
CIR	uint16_t[]	CIR data

## Data Availability

Not applicable.

## Abbreviations

The following abbreviations are used in this manuscript:

AP	access point
CIR	channel impulse response
COTS	commercial off-the-shelf
CSI	channel state information
CQI	channel quality indicator
DFL	device-free localization
ECDF	empirical cumulative distribution function
MA	multipath-assisted
MPC	multipath component
MQTT	message queueing telemetry transport
RF	radio frequency
RSS	receive signal strength
RTI	radio tomographic imaging
UWB	ultra-wideband
